# Does the Diffusion Profile Differ Between Botulinum Toxin Type a Formulations? Implications for the Management of Post-Stroke Spasticity

**DOI:** 10.3390/toxins16110480

**Published:** 2024-11-07

**Authors:** Alessandro Picelli, Stefano Tamburin, Rita Di Censo, Nicola Smania, Mirko Filippetti

**Affiliations:** 1 Department of Neurosciences, Biomedicine and Movement Sciences, University of Verona, 37134 Verona, Italy; stefano.tamburin@univr.it (S.T.); rita.dicenso@univr.it (R.D.C.); nicola.smania@univr.it (N.S.); mirko.filippetti@univr.it (M.F.); 2 Department of Neurosciences, University Hospital of Verona, 37126 Verona, Italy; 3 Canadian Advances in Neuro-Orthopedics for Spasticity Consortium (CANOSC), Kingston, ON K7K 1Z6, Canada

**Keywords:** botulinum toxins, muscle spasticity, therapeutics

## Abstract

Botulinum toxin type A is a first-line treatment for post-stroke spasticity, with selective action at nerve endings and minimal effects beyond the injection site. However, concerns about potential adverse reactions due to toxin diffusion and spread can significantly influence physicians’ therapeutic decisions in managing post-stroke spasticity. Current evidence shows that while the main formulations of botulinum toxin type A have different molecular weights and sizes, they do not exhibit differing diffusion profiles. Instead, the key factors determining botulinum toxin type A diffusion and spread in post-stroke spasticity management are the dose (i.e., the actual amount of 150 kDa neurotoxin protein injected), dilution, and injection volume. Other injection-related factors, such as the needle gauge and injection speed, have also been suggested to have a secondary influence on botulinum toxin type A diffusion and spread. The needs of patients with post-stroke spasticity may vary, and depending on treatment goals, botulinum toxin type A diffusion and spread can be something to avoid or may offer therapeutic benefits by reaching a greater number of nerve terminals in the target muscle, enhancing the toxin’s effect. These factors should be carefully evaluated in spasticity clinics.

## 1. Botulinum Neurotoxins: Serotypes and Mechanism of Action

Botulinum neurotoxins (BoNTs) are produced by neurotoxigenic strains of bacteria belonging to the genus *Clostridium* [1,2]. To date, eight serotypes of BoNTs have been identified and labeled with the letters A, B, C, D, E, F, G, and X [1]. All BoNT serotypes share a similar molecular architecture, consisting of a single-chain polypeptide of 150 kDa [1,2]. The toxin active protein consists of a light (L) chain (50 kDa) and a heavy (H) chain (100 kDa) connected by a disulfide bond [1,2,3].

The C-terminal of the H chain mediates the specific, rapid, and strong interaction of BoNTs with the unmyelinated areas of peripheral cholinergic nerve endings [1,2,3]. This binding triggers the subsequent internalization and trafficking of BoNTs within endocytic compartments, initiated by the retrieval of synaptic vesicles following the release of their neurotransmitter content [2]. The amino-terminal domain of the H chain plays a crucial role in translocating the L chain across the endocytic vesicle membrane into the cytosol [2,3]. The VAMP/synaptobrevin, SNAP-25, and syntaxin proteins form complexes (SNARE) that mediate vesicle docking and membrane fusion to release neurotransmitters into the synaptic cleft [1,2,3]. The SNARE proteins serve as specific substrates for the catalytic activity of the L chain metalloprotease, which selectively cleaves them at different peptide bonds, thereby inhibiting neurotransmitter release into the synaptic cleft [1,2].

## 2. Application of Botulinum Neurotoxins in Spasticity Clinics

Research into the potential clinical use of BoNTs began in the 1970s, with pioneering studies conducted by Alan Brown Scott and colleagues [4]. In 1989, the Food and Drug Administration approved the clinical use of BoNT/A for the first time as a treatment for blepharospasm and strabismus in adults [4,5]. Today, BoNT/A and BoNT/B are widely used in clinical practice for various therapeutic indications, with new formulations currently under development [1,5].

Only BoNT/A has been approved for the treatment of post-stroke spasticity (PSS) [4,5,6,7]. Regarding its mechanism of action, BoNT/A selectively targets SNAP-25, generating cleavage fragments (SNAP-251-197) that inhibit acetylcholine exocytosis at the neuromuscular junction, leading to a neuromuscular blocking effect [1,2,3]. The duration of BoNT/A action is approximately 3–4 months in humans, so toxin injections are typically repeated in cycles as long as patients continue to benefit [1,2,6].

Currently, three major branded products containing BoNT/A are commercially available worldwide for the treatment of PSS: onabotulinumtoxinA, marketed as Botox by Abbvie Inc. (North Chicago, IL, USA); abobotulinumtoxinA, marketed as Dysport by Ipsen (Boulogne-Billancourt, France); and incobotulinumtoxinA, marketed as Xeomin by Merz Pharmaceutical GmbH (Frankfurt, Germany) [8]. As shown in Table 1, these products have different characteristics and should not be considered interchangeable [9].

## 3. Discussion

The potential for both local and systemic adverse events is a major concern in the management of PSS with BoNT/A [10]. Toxin diffusion and spread are believed to account for most of the local, distal, and systemic side effects of BoNT/A therapy [10,11]. In particular, diffusion specifically involves a microscopic phenomenon, in which a soluble molecule is passively transported beyond its original injection site [10,11,12]. The diffusion of unbound toxin through extracellular space causes spread, which refers to the physical movement of the toxin from one site to another [11,12].

The early literature on the clinical use of BoNT/A reports a significantly greater diffusion profile for abobotulinumtoxinA compared to onabotulinumtoxinA [13]. Based on these findings, protein composition and molecular size were suggested to influence toxin diffusion, with larger proteins moving more slowly through the same aqueous medium than smaller ones [2,10,12,13]. As a result, it was hypothesized that BoNT/A formulations with higher molecular weights or larger sizes would be less likely to diffuse beyond the target muscle [12,13]. In this context, onabotulinumtoxinA (complex size 900 kDa) was expected to diffuse less than abobotulinumtoxinA (complex size ~500 kDa) [13]. However, more recent clinical evidence contradicts this assumption, showing no differences in diffusion between onabotulinumtoxinA (complex size 900 kDa) and incobotulinumtoxinA (complex size 150 kDa) [10,11,14]. Furthermore, evidence from animal models does not support considering protein composition and molecular size as key factors determining BoNT/A diffusion [11]. In particular, Carli and colleagues conducted a thorough assessment of the diffusion of the three major commercial BoNT/A preparations using a highly sensitive test based on the expression of the neural cell adhesion molecule (N-CAM), a membrane glycoprotein that accumulates on muscle fibers after denervation and is absent in untreated adult muscle [15]. Following toxin injections into the tibialis anterior muscle of each hind limb, immunohistochemical analysis of N-CAM expression revealed no significant differences in diffusion between abobotulinumtoxinA, incobotulinumtoxinA, and onabotulinumtoxinA into muscles adjacent to the tibialis anterior in the mouse leg [15].

Conversely, the current literature indicates that dosage, along with dilution and injection volume, are main issues as to the diffusion of BoNT/A [10,11,12]. According to American and Italian labeling information, the maximum dose per treatment session for adults with PSS should not exceed 400 units of onabotulinumtoxinA, 1500 units of abobotulinumtoxinA, and 500 units of incobotulinumtoxinA [16,17]. These limits are higher compared to those for other conditions, such as visual disturbances, dystonia, or cosmetic treatments. This is because the treatment of PSS requires injections into large skeletal muscles in the upper and lower limbs, such as the brachialis, quadriceps, and triceps surae [18]. The same applies to dilution, which should be higher for muscles with large volumes or diffuse motor endplates, where multiple injection sites are also recommended [18,19]. Therefore, the dosage, concentration and injection volume of BoNT/A are of greater concern in PSS than other indications.

In our view, some of the controversies surrounding the (supposed) differences in diffusion profiles among the major BoNT/A preparations in clinical settings may be attributed to inappropriate means of comparison. When focusing solely on the 150 kDa neurotoxin protein, the average amount of BoNT/A per vial is 2.69 ng/500 units of abobotulinumtoxinA, 0.40 ng/100 units of incobotulinumtoxinA, and 0.90 ng/100 units of onabotulinumtoxinA [20]. So, according to current labeling information, the maximum recommended dose per treatment session for PSS in adults corresponds to 8.07 ng (1500 units; 3 vials × 500 units) of abobotulinumtoxinA, 2.00 ng (500 units; 5 vials × 100 units) of incobotulinumtoxinA, and 3.6 ng (400 units; 4 vials × 100 units) of onabotulinumtoxinA [17,20]. This implies that in a multi-level, multi-pattern treatment of PSS using the maximum recommended total dose of BoNT/A, the same patient could receive up to four times the amount of BoNT/A depending on the preparation injected. Such a discrepancy inevitably affects the methods used to assess the extent of local diffusion and toxin spread (e.g., N-CAM expression by muscles adjacent to the injected ones) [15], as well as the occurrence of adverse events, which may be mistakenly attributed to differences in diffusion profiles. In reality, the issue probably would relate to dosage, a key determinant of toxin diffusion [10,11,12]. Our hypothesis seems to be confirmed by studies that report similar diffusion profiles between BoNT/A formulations when a conversion ratio of 1:4 is applied between incobotulinumtoxinA and abobotulinumtoxinA and a ratio of 1:0.75–0.5 is considered between incobotulinumtoxinA and onabotulinumtoxinA [15,21]. Additionally, our argument is further supported when dilution and injection volume (the other two key factors influencing toxin diffusion and spread) are seen in light of these conversion ratios. In fact, when looking at the typical dilutions used in daily clinical practice for the management of PSS in adults (100 U/2 mL for onabotulinumtoxinA and incobotulinumtoxinA; 500 U/2.5 mL for abobotulinumtoxinA), the concentration ratio is four time greater for abobotulinumtoxinA (1.076 ng/mL) and double for onabotulinumtoxinA (0.45 ng/mL) than incobotulinumtoxinA) (0.2 ng/mL). Other injection-related factors, such as needle gauge and injection speed, have also been suggested to have a secondary influence on BoNT/A diffusion and spread. Indeed, larger needles and faster injections may cause trauma to the target tissue, potentially reducing toxin uptake at the injection site and increasing diffusion into adjacent areas [10,11].

## 4. Implications for the Management of Post-Stroke Spasticity

The needs of patients with PSS may vary. For example, patients with low functional upper limb and severe clenched fist usually require high-dose BoNT/A injections into the finger flexor muscles to meet posture and hygiene goals. In these patients, there is far less need for selective injections into the finger flexor fascicles than individuals with focal hand dystonia, and local diffusion of BoNT/A (i.e., spread into the muscle fascicles near to the injected ones) is considered desirable in order to obtain a greater effect. Conversely, in stroke patients with a good functional profile who suffer from upper limb spastic dystonia, toxin diffusion may represent an unwanted effect.

Many clinicians attribute some of the local and systemic adverse events in adult PSS patients treated with BoNT/A to the different diffusion profiles of BoNT/A preparations. Actually, this issue seems to be largely procedural, as dosage, dilution, and injection volume are recognized as the key factors influencing diffusion and toxin spread, rather than the specific characteristics of different products (i.e., protein composition and molecular size).

From a pharmacological perspective, the optimal effect is achieved when BoNT/A reaches the maximum number of nerve terminals innervating the target muscle [2]. Therefore, toxin local diffusion within the muscle is not always something to avoid. In fact, some clinicians may leverage local diffusion effects when treating PSS with higher dilution volumes of BoNT/A, particularly when they cannot increase the total dose or need to inject large spastic muscles [10,11,16,17]. This approach can enhance the distribution of the toxin to neuromuscular junctions further from the injection site, resulting in greater muscle tone reduction [2]. Likewise, multi-site injection may further help to improve toxin distribution and diffusion into the treated muscles.

From a procedural standpoint, the amount of BoNT/A injected and its concentration should not follow a standard approach but should be based on the actual quantity of toxin injected (i.e., ng of 150 kDa protein) and the characteristics of target muscles [20]. Current best practices in PSS recommend using higher doses, multiple injection sites, and lower concentration volumes for larger muscles (e.g., biceps brachii and triceps surae) [18,19]. Conversely, low dilution volumes and one or few injection sites are recommended for smaller muscles (i.e., higher doses do not necessarily require higher dilution volumes, and higher dilution volumes do not imply larger injection volumes) [18]. Additionally, current evidence strongly supports the use of instrumented guidance for BoNT/A injections in the treatment of PSS [22,23,24,25]. Ultrasound guidance, in particular, allows for the assessment of muscle anatomy, size, and structure, as well as the real-time visualization of the injection site, needle placement, and BoNT/A distribution along the muscle fibers [26,27,28]. Moreover, it may be helpful for overcoming other issues related to the injection procedure, such as the choice about the needle gauge and the regulation of the injection speed. Electromyography-guided injections may further assist in targeting the motor end-plate region [29].

In conclusion, while the diffusion profiles of different BoNT/A formulations do not appear to be a major concern in the treatment of PSS, key factors such as dosage, dilution, and injection volume, along with other procedural considerations, should be carefully evaluated based on each patient’s specific situation. This is because BoNT/A diffusion and spread can potentially offer also therapeutic benefits in certain clinical scenarios. Adopting this approach may help to prevent adverse events when planning BoNT/A treatment for PSS [30].

## Figures and Tables

**Table 1:** The main characteristics of branded products containing BoNT/A [8].

	AbobotulinumtoxinA	IncobotulinumtoxinA	OnabotulinumtoxinA
Brand name	Dysport	Xeomin	Botox
Units per vial	300; 500	50; 100; 200	100; 200
Complex size	~500 kDa	150 kDa	900 kDa
Preparation	Lyophilized	Lyophilized	Vacuum-dried
Storage	2–8 °C	<25 °C	2–8 °C
Constituents and excipients	Hemagglutinin, human albumin, lactose	Human albumin, saccharose	Hemagglutinin, human albumin, saccharose, NaCl

## Data Availability

No new data were created or analyzed in this study.
